# N-Doped Mesoporous Carbons: From Synthesis to Applications as Metal-Free Reduction Catalysts and Energy Storage Materials

**DOI:** 10.3389/fchem.2019.00761

**Published:** 2019-11-12

**Authors:** Ying Yang, Lin Gu, Shangwei Guo, Shuai Shao, Zelin Li, Yuhang Sun, Shijie Hao

**Affiliations:** State Key Laboratory of Heavy Oil Processing, China University of Petroleum, Beijing, China

**Keywords:** N-doping, mesopore, synthesis, catalysis, energy storage

## Abstract

N-doped mesoporous carbons, NMCs, have attracted intensive attention recently and have shown potential applications in various scientific fields including catalysis and energy conversion/storage. Via modification with foreign N elements and construction of mesoporous structures for NMCs, their electronic and spin structure, as well as their porosity can be greatly tailored. And the resultant electron-donor property, surface wettability, conductivity, ion/molecular transfer and reactivity are changed accordingly. In this review, we will summarize the recent research progress of these metal-free NMCs, with an emphasis on their synthesis and performance, especially for their synthetic strategy and catalytic properties toward oxygen and nitro compound reductions, as well as their electrochemical properties as electrode materials for lithium-ion/sulfur batteries and supercapacitors. We hope for future developments, such as controlling doping methods more precisely, generating more active sites by N-doping, and finding wider applications of NMCs in other fields.

## Introduction

Carbon materials, due to their unique physical and chemical features, have been widely used as catalyst carriers (Rodríguez-reinoso, 1998; Joo et al., 2001), adsorbents (Xiang et al., 2013; Gong et al., 2017), water treatment catalysts (Liang et al., 2011; Wang et al., 2017), and electrode materials for energy conversion/storage (Li et al., 2012; Dutta et al., 2014; Yang T. et al., 2015). Although various nanostructured carbons have been investigated, their performances are still not able to satisfy the increasing demand for catalysis and energy conversion/storage applications. Doping carbons with N heteroatoms proves to be efficient to improve the performance of nanostructured carbons (Böttger-Hiller et al., 2012; Han et al., 2013; Zhang et al., 2013; Lin et al., 2015). N-doped carbons, regarded as “noble carbons,” can largely improve the surface wettability, electron-donor property, conductivity and reactivity of carbons, even a small amount (<0.5%) of N atoms are incorporated (Zhou et al., 2018). The lone-pair electrons of doped N atoms can provide additional negative charges, leading to the improved physicochemical properties and strong interactions with exotic species (i.e., acidic compounds, cationic ions, and metal nanoparticles) (Xu et al., 2012; Li et al., 2013). These characteristics play a significant role in many challenging applications, especially in the field of catalysis and electrochemistry. Nevertheless, the performance of N-doped carbon materials are not only related to the N moieties, but also correlated with the access of reactants to the active sites. In this regard, NMCs with accessible mesopores (2–50 nm), are preferred because the mass transport and the charge/ion transfer are faster than those with microporous carbons involved (Jaouen et al., 2006; Daems et al., 2014). NMCs are therefore of particular interest recently, because of their large specific surface area and adjustable mesopores (Liang et al., 2008; Zhang et al., 2017). And construction of NMCs combined with rich N dopants and mesoporous structure is urgently desirable for catalysts, fuel cells, and components of electrodes.

Achieving the effective N introduction and mesopore construction is highly dependent on the synthetic strategy. Post-treatment of carbon materials with ammonia or N-containing precursors (Roy et al., 1997; Jiang and Gao, 2003; Sidik et al., 2006; Titirici et al., 2007; Shao et al., 2008; Zheng et al., 2012), as well as *in situ* pyrolysis of N-containing compounds, is used to prepare N-doped carbons. Since the latter can generate uniformly incorporated, dense N dopants throughout the carbonaceous skeleton, various N-containing precursors, such as melamine (Terrones et al., 1999a,b), benzylamine (Terrones et al., 2000), acetonitrile (Glerup et al., 2003; Kudashov et al., 2004), N-heterocycles (Sen et al., 1997; Liu et al., 2005) and phthalocyanines (Zhi et al., 2005), as well as biopolymeric N-containing precursors like silk (Iwazaki et al., 2010), pulse flour (Gokhale et al., 2014), honey (Lu et al., 2013), hair (Chaudhari et al., 2014), and egg white (Wang et al., 2013), are involved in one-pot N-doping. On the other hand, to construct the mesoporous structure, the templating method is commonly employed. Compared to the tedious and expensive hard-templating approach (Silva et al., 2013; Wei et al., 2014; Niu et al., 2016; Wan et al., 2016), soft-templating approach is more facile even at a scalable production, since the pre-synthesis of templates is not necessary, and the organic templates can be removed during pyrolysis (Liang et al., 2004; Liu J. et al., 2013). However, pyrolysis at a high temperature can speed up the break of N-containing networks, and the resultant N content is declined and the mesoporous structure is collapsed after template removal (Moreno et al., 2014; Qiang et al., 2017). On the other hand, direct carbonization of N-containing ionic liquids, citrate salts or sulfuric acid-dehydrated sucroses can yield NMCs with either wide and disordered mesopores (Fellinger et al., 2012; Ferroro et al., 2016; Wu et al., 2017), or microporous structures (Zhu et al., 2013; She et al., 2015). Although several NMCs were facilely fabricated for catalytic reduction reactions, Li-ion batteries and fuel cells, by direct pyrolysis of metal-organic framework (MOF) and covalent organic framework (COF), such as ZIF-8, zinc-dicarboxylic acid containing MOF (NZnMOF), and hydrogen-bonded organic framework (HOF) (Chaikittisilp et al., 2012; Yang et al., 2012; Yang T. et al., 2015; Liu C. et al., 2016). It is still very challenging yet desirable to facilely fabricated NMCs within homogeneous/rich N dopants and well-defined mesopores. We noticed that the relative research about NMCs is still in its infancy. Synthesis of N-doped graphene and carbon nanotube, and their performance in oxygen reduction reactions, have been reviewed (Yu et al., 2010; Kong et al., 2014). Whereas, the mesoporous structure of N-doped carbons and their applications in other fields have never been focused.

In this paper, we will overview the latest progress of NMCs in the catalytic chemistry and electrochemistry, as promising metal-free catalysts and energy storage materials, with the hope of stimulating further exploration of their synthesis and applications in various reactions. It is important to note that this review only focuses on pure NMCs, and thereby graphitic carbon nitrides (g-C_3_N_4_) with dense N moieties, and composites like NMC-carbon and NMC-metal are ignored. The report of different NMCs is introduced according to the type of reactions they are involved in. In each reaction section, the existing synthesis method will be briefly introduced, and readers can refer to previous studies and obtain more detailed information if necessary. Then, the N dopants and mesopore characteristics and their roles in catalytic reduction reactions and energy conversion/storage will be reviewed. Finally, a summary and an outlook on the future development of NMCs will be presented.

## Reduction Reaction Catalysts

### Oxygen Reduction Reaction

**Figure 1:**
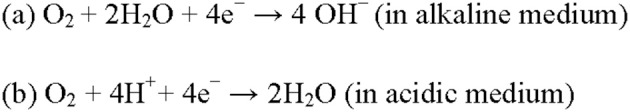
The four-electron ORR process in **(a)** alkaline and **(b)** acidic medium.

The electrochemical reduction of oxygen is highly relevant in metal-air batteries, fuel cells and air-breathing cathodes. However, poor kinetics, expensive Pt catalysts and CO poisoning are the primary factors retarding the implementation of relevant devices. To reduce or replace platinum-based electrodes, N-doped carbon nanostructures, including N-doped carbon nanosphere, nanotube and graphene, have been developed and proved to be extremely effective as metal-free ORR electrocatalysts. These N-doped carbons are used to catalyze a four-electron ORR process (Figure 1), which exhibit superior electrocatalytic activity and operational stability than Pt-based catalysts.

**Figure 2:**
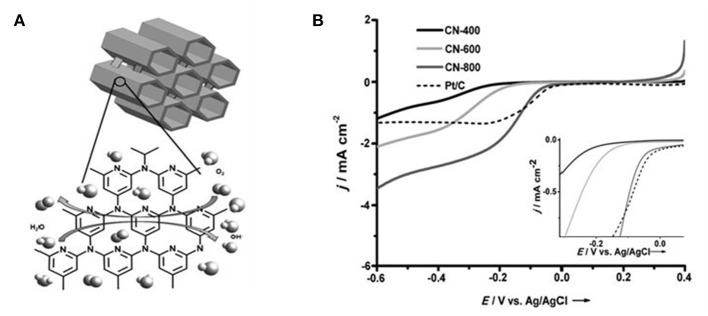
**(A)** Schematic illustration of N-doped ordered mesoporous graphitic array and N-doped ordered mesoporous carbon synthesis. **(B)** Polarization curves of MNC catalysts pyrolyzed at different temperatures in 02-saturated 1 M NaOH with a rotation rate of 900 rpm at a scan rate of 5 mV s^−1^ CE: Pt grid, RE: AglAgCl/3M KCl (inset: magnification of the potential region of the ORR onset). Reprinted from Vinu (2008), with permission from Wiley.

Recent investigations about the N-doped carbon nanotube and graphene ORR catalysts are mainly focused on the content and the type of N dopants, as well as their relationship with the ORR properties (Geng et al., 2011). It is found that N-doping level affects the content of different N types, which usually varies with the situation. Adding more N species does not mean better performance, but also depends on the pore structure (Matter et al., 2006). To facilitate the diffusion of oxygen and electron/ion transfer especially under a slow stirring rate, NMCs with well-defined mesopores were then fabricated. In primitive investigations, by a metal-free hard-templating strategy, N-doped ordered mesoporous graphitic arrays (Liu et al., 2010) and N-doped ordered mesoporous carbons (specific surface area, 1500 m^2^ g^−1^) (Wang et al., 2010) were fabricated using N-containing aromatic dyestuff and polyaniline as the carbon precursor, respectively (as illustrated in Figure 2A). The results show that the obtained NMC has a very high catalytic activity for ORR. Specifically, compared with commercial 20 wt% Pt/C, these NMCs have lower ORR starting voltage and higher methanol tolerance in alkaline/acidic media. Nagaiah et al. reported the nanocasted, mesoporous N-rich carbons prepared by pyrolysis of ethylenediamine-containing SBA-15 at different temperatures, followed by silica removal (Vinu, 2008). Interestingly, with the increase of pyrolysis temperature, the surface area and pore volume increase. Accordingly, the positively shifted onset potentials and the increased current density at lower potentials are observed (Figure 2B). Besides the N-doping, the mesostructure is also responsible for the enhanced activity. Either an active site limitation of the current density for Pt/C or a lower average number of electrons transferred per O_2_ molecule was observed. The mesoporous structure of NMC materials enhanced the current density at lower potentials as well as the less steep current decrease in the kinetic region of the polarization curve, that increased the probability of re-adsorption of primarily formed hydrogen peroxide and consecutive electron transfer steps.

**Figure 3:**
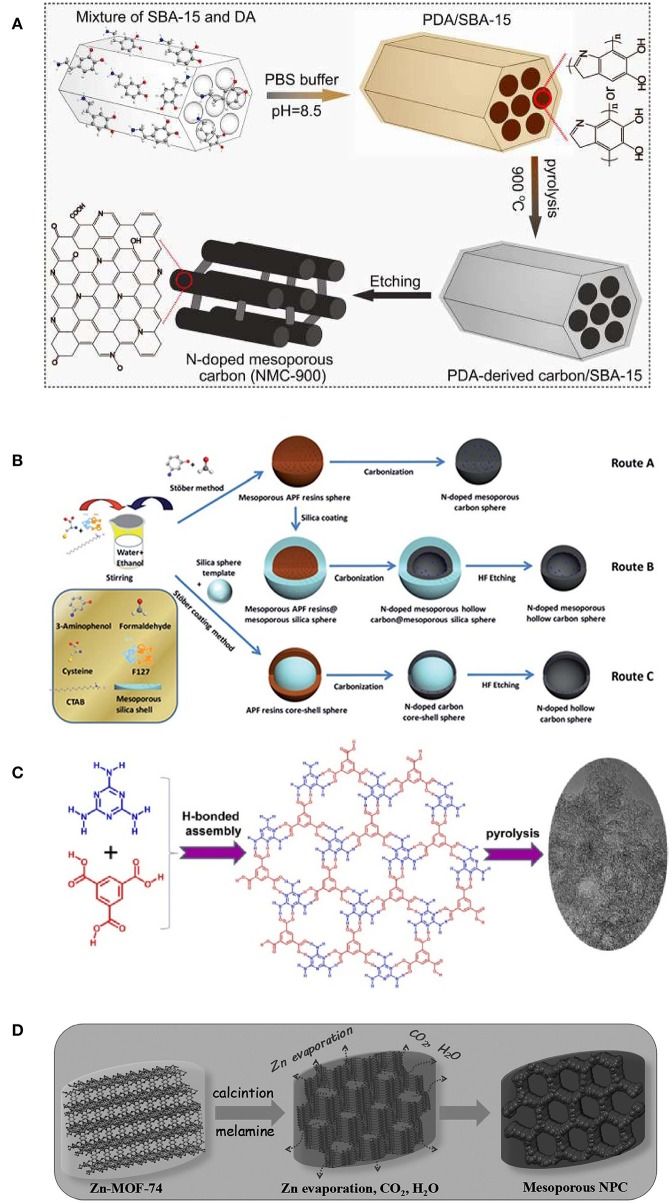
**(A)** Fabrication of PDA-derived NMC-900. From Qu et al. (2018) with permission from Wiley. **(B)** Experimental routes were taken to prepare the N-doped carbon spheres in different nanostructures (The blue dots represent the N doping). Route A: nitrogen-doped mesoporous carbon nanospheres (N-MCNSs); route B: nitrogen-doped mesoporous hollow carbon nanospheres (N-MHCNSs); and route C: nitrogen-doped hollow carbon nanospheres (N-HCNSs). From Yang T. et al. (2014) with permission from Royal Society Chemistry. **(C)** The process of preparation of N-doped mesoporous carbon from HOF. From Liu C. et al. (2016) with permission from Wiley. **(D)** The process of preparation of the nitrogen-doped mesoporous carbon. From Ye et al. (2017) with permission from Wiley.

Besides one-dimensional NMCs, templating synthetic strategies have been also developed to fabricate three-dimensional (3-D) NMCs with interconnected channels, that can accelerate the mass transfer during synthesis (Figure 3A). The 3-D structured NMCs display high catalytic efficiency in ORR and excellent stability greatly exceeds that of Pt/C, owing to its fascinating composition and structural advantages. Graphitic N and pyridinic N are efficient and stable metal-free active sites. Large surface area and well-arranged 3-D mesoporous structure provide fully exposed active sites and unobstructed reaction pathways for ORR (Qu et al., 2018). Raman spectra and XPS studies demonstrate that the NMC-900 (pyrolyzed at 900°C) gives higher electrical conductivity, and the hydrophilic surfaces originated from abundant O groups make exposed active sites readily available, both accelerating ORR kinetics.

The contact between oxygen and catalysts can be improved as nanostructured NMC spheres were utilized. Specifically, their spherical shape and large pore volume can improve the activity of electrocatalysts by ensuring that the reactants are easily accessible to the catalytic active sites and the mass transfer is rapid. Through a simple and low-cost dual-soft templating strategy, Qiao's group designed N-doped mesoporous carbon spheres using resorcinol formaldehyde resin/melamine as the carbon/nitrogen precursor (Bayatsarmadi et al., 2015), and formaldehyde/3-aminophenol (Figure 3B, route A) as the carbon/nitrogen source (Yang T. et al., 2014), respectively. To introduce more porosity, a silica-assisted coating method was employed to N-doped mesoporous hollow carbon nanospheres (N-MHCNSs) with tailored shell thickness, pore size and surface area (Figure 3B, route B), and doped hollow carbon nanospheres (N-HCNSs, Figure 3B, route C) (Yang T. et al., 2014). From the high kinetic current and positive onset potential, all the resultant NMC spheres are proved to be highly active for ORR in alkaline solutions. In particular, the N-doped mesoporous hollow carbon sphere with particle size of ca. 150 nm, exhibited the highest activity. This can be ascribed to the enhanced interaction and dissociation of oxygen within the pores and on the surface, the improved wettability to electrolyte solution and the charge/spin density on the carbon.

Sheet-like NMCs that can expose more active N dopants, are much favored and were fabricated as ORR catalysts by thermal decomposition of low-cost precursors. It is known that chitosan is obtained as the N-deacetylated derivative of chitin, comes from waste shells from crustaceans such as shrimps and crabs, while melamine is a very inexpensive industrial raw material that contains rich N moieties. By conversion of chitosan-melamine precursor, the resultant NMC comprises lots of nanosheets, having a specific surface area (*2*85 m^2^ g^−1^), are highly efficient for ORR in alkaline media (Rybarczyk et al., 2015). This strongly suggests that the sheet-like morphology can improve the accessibility of active sites.

More recently, direct conversion of N-containing crystalline HOF and MOF emerges as a brand-new strategy to prepare NMCs. Considering the rich N content of melamine and its easy assembly with trimesic acid to form HOF, the NMC was prepared by pyrolysis of HOF precursors (Figure 3C). The HOF-derived NMC exhibits a superior electrocatalytic activity for ORR compared with Pt/C catalyst. It is noteworthy that the introduction of high content graphitic N into the carbon skeleton improved the conductivity of graphene sheets, that facilitates the electron transfer and hence promotes the ORR activity (Liu C. et al., 2016). Wen et al. recently reported an effective and facile route to fabricate NMCs by pyrolysis of Zn-MOF-74 (Figure 3D). During heating, the Zn-MOF-74 was molten and decomposed, and mesopores were formed as the collapse of pristine frameworks occurred. This process was accompanied by Zn metal removal when the temperature reached the boiling point of Zn (ca. 908°C), which is beneficial to produce more porous structures. As expected, Zn-MOF-74 derived NMC is highly active and stable, as an efficient pH-universal electrocatalyst for ORR. Density functional theory calculation demonstrates the special N dopants and easily accessible pores in the NMC that lead to the largely improved activity synergistically. And the introduction of N dopants can tailor the electronic properties and conductivity of NMC, that leads to the remarkably enhanced ORR activity (Ye et al., 2017).

### Electrochemical Synthesis of Hydrogen Peroxide

**Figure 4:**
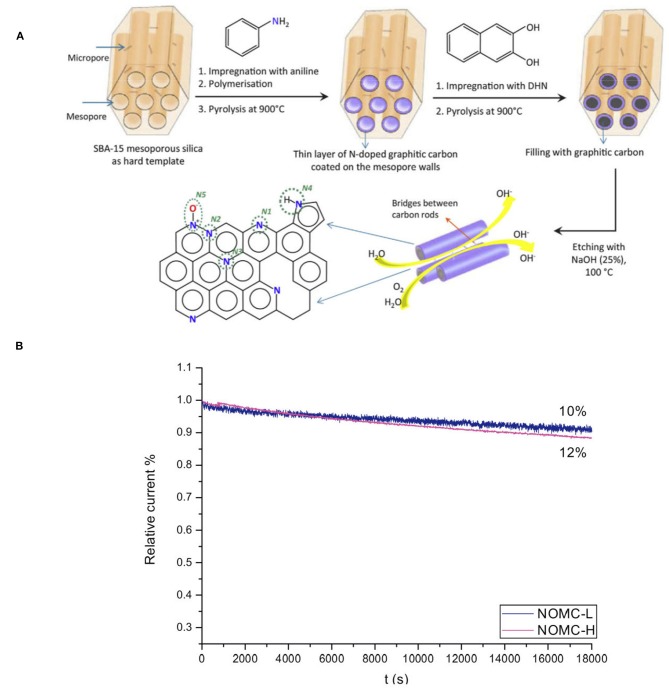
**(A)** Schematic illustration of the formation of a NOMC with a layer of N-doped carbon at the surface and a fully carbonaceous core, using SBA-15 as hard template. The possible configurations of N atoms in the NOMC material are indicated: pyridinic N (N1), graphitic N (N2-N3), pyrrolic N (N4), and oxidized pyridinic N (N5). **(B)** Relative current density vs. time (J-t) plot based on chronoamperometric measurements of NOMCs at −0.4 V vs. Ag/AgCl in O_2_-saturated 0.1 M KOH at a rotation speed of 1,600 rpm. From Sheng et al. (2015) with permission from ScienceDirect.

Hydrogen peroxide (H_2_O_2_) is a widely used chemical in various fields, including use as the green oxidant (Jones and Clark, 1999). However, its standard production through the anthraquinone process suffers from high cost and hazardous nature. Therefore, an environmental benign process using hydrogen and oxygen as reactants is very ideal. The electrochemical method has the advantages of oxygen reduction and hydrogen oxidation in two separate cells, thus avoiding the direct contact and making the process essentially safer. Although continuous research on improving the electrochemical synthesis of H_2_O_2_ was reported (Otsuka and Yamanaka, 1990; Gyenge and Oloman, 2001; Lunsford, 2003; Choudhary and Jana, 2007; Forti et al., 2007; Lobyntseva et al., 2007; Samanta and Choudhary, 2008; Jirkovsky et al., 2011), the involved electrocatalysts are expensive, and show limited selectivity or low activity. In this regard, Antonietti et al. designed a cheap mesoporous N-doped carbon (16.2%), that was synthesized from an ionic liquid, N-butyl-3-methyl pyridinium dicyanamide, and was proved to be highly active and selective for the electrochemical synthesis of H_2_O_2_ (Fellinger et al., 2012). It is reported that the electrocatalytic activity of such a NMC is generally attributed to the higher N electronegativity as compared to C, that leads to a polarization of the C-N bonds, thus altering the electronic property of the carbon material and creating sites for interaction with O_2_. Later, N-doped ordered mesoporous carbon (NOMC) was designed as an efficient catalyst for the electrochemical reduction of O_2_ to H_2_O_2_. NOMC was prepared by pyrolysis of aniline and dihydroxynaphthalene that were confined into SAB-15, showing a high surface area (877 m^2^ g^−1^) (Figure 4A). The NOMC proves to be highly selective toward the reduction of O_2_ to H_2_O_2_, and is extremely stable in an acidic media (Figure 4B), following the reaction O_2_ + 2H^+^ + 2e^−^ → H_2_O_2_ (Sheng et al., 2015). It is considered that the incorporation of N into the carbon material enhances its electron-donor properties, and the increase of N content, as well as the “free radical” characteristics of nitrogen-bound carbon, leads to the enhanced electrochemical activity. Though both graphitic N and pyridinic N have contributed to the electrocatalytic activity of NOMC, the exact role of these two types of sites in the reaction mechanism and their influence on the selectivity toward the partial reduction to H_2_O_2_ is still a matter of debate.

### Nitro Compound Reduction

Nitro compounds, like 4-nitrophenol (4-NP) and nitrobenzene (NB), are common organic pollutants in agricultural and industrial production (Nemanashi and Meijboom, 2013). The conversion of nitro group to amino moiety shows great industrial significance, such as the preparation of aniline and paracetamol (Fenger et al., 2012). Noble metal catalysts like Au, Ag, and Pd, along with alloys like PtNi, AuCu, and PdCu (Lin et al., 2012; Pozun et al., 2013; Yang Y. et al., 2014), were found to be efficient to convert nitro compounds. Considering high price of precious metals and the secondary pollution caused by the metal leaching, metal-free catalysts clearly emerge as new and promising candidates for the reduction of nitro compounds. It is reported that a resin-supported dye was active toward the photocatalytic reduction of 4-NP under visible-light irradiation (Gazi and Ananthakrishnan, 2011). N-doped graphene (NG) exhibited a high activity comparable to that of metal catalysts toward 4-NP reduction (Kong et al., 2013). However, these N-doped catalysts often have low surface area and are mainly microporous, that is adverse to the substrate diffusion and mass transfer during reduction reactions.

**Figure 5:**
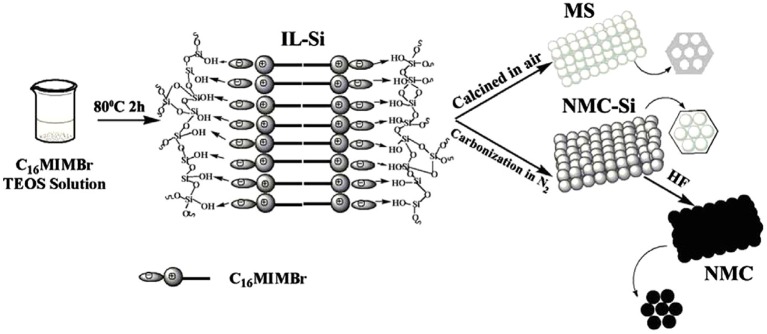
Schematic of the formation process of NMC and MS. From Liu C. et al. (2013) with permission from ScienceDirect.

**Figure 6:**
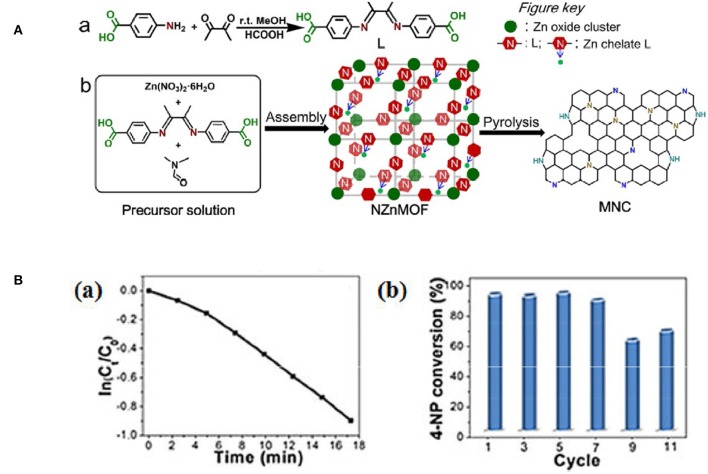
**(A)** Schematic illustration of (a) the synthesis of 1,4-bis(4-C02HC6H4)- 2,3-dimethyl-1,4-diazabutadiene (L) and (b) the synthesis of mesoporous N doped carbons through pyrolysis of N-rich MOF precursors. From Yang T. et al. (2015) with permission from Wiley. (**B**,a) The relationship between ln(Ct Co) and reaction time (t), and (b) catalytic conversion of 4-NP at different cycles. From Yang T. et al. (2015) with permission from Wiley.

NMCs have large surface area and regular mesoporous arrangement, that makes active N dopants more accessible. Chen's group reported the N-doped mesoporous carbon (3.2%) that was synthesized using ionic liquid (IL) as the carbon precursor (Figure 5). The obtained NMC shows superior catalytic performance for the NB reduction with 88.6% yield of aniline (Liu C. et al., 2013). Our group developed a new NMC by direct conversion of N-containing metal-organic frameworks (NZnMOF) (Figure 6A). The doped N species are homogeneously dispersed on mesoporous carbons, and additional carbon/nitrogen source and structure-directing template are not necessary. Interestingly, the NMC involved catalytic process was found to be zero order kinetics (Figure 6Ba), that is distinguished from the first order kinetics as metal catalysts were utilized. And a much larger specific rate constant was displayed as compared to that as NG was involved, and a long-term stability (up to 11 times) can be observed (Figure 6Bb). Further N elemental analysis and XPS investigations demonstrate that the activity does not depend on the amount of N dopants, but on the total amount of graphitic N that provides appropriate adsorption sites. The superior activity of NMC, as compared to NG, is originated from the enhanced content of active N sites (50.1%). It is reasonable that the thermal decomposition at a high temperature (950°C) may facilitate the conversion of amine N to graphitic N. Compared to traditional N-doped carbons, our NMC bears a higher accessible surface area and a larger mesopore, which can facilitate the substrate diffusion and mass transfer. So the mesoporous network and effective N-doping could improve the product diffusion and 4-NP adsorption/activation, leading to an excellent performance (Yang Y. et al., 2015).

## Energy Storage Materials

### Lithium-ion/Sulfur Batteries

**Figure 7:**
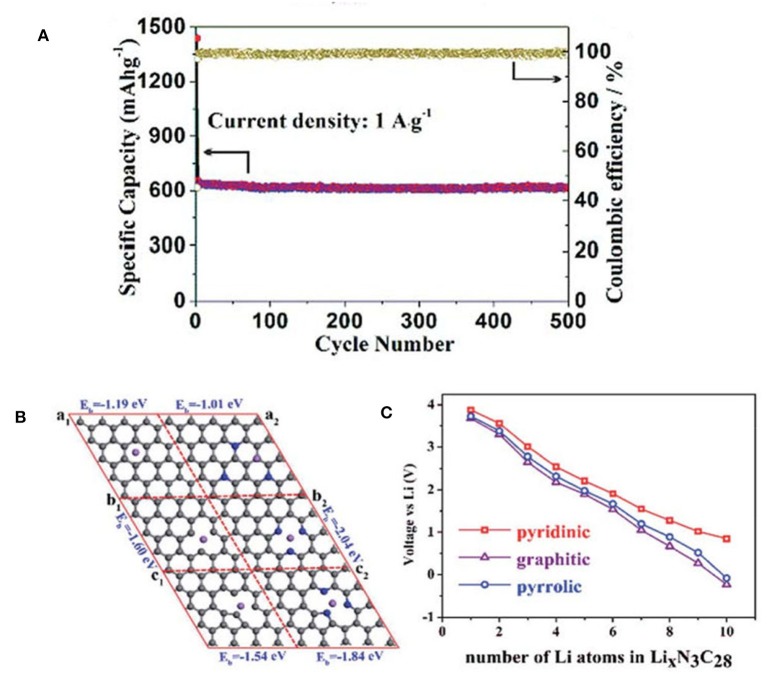
**(A)** Nitrogen-doped carbon honeycomb-like structure assembled by two-dimensional mesoporous nanosheets were successfully fabricated via a thermal annealing and acid etching strategy. The sample exhibited excellent electrochemical properties as anode materials for LIBs. **(B)** Comparison of three kinds of C stable sites before and after doped N after Li atom adsorbed (al-pure Graphene, a2-N doped Graphene, bl-pure pyridinic, b2-N doped pyridinic, cl-pure pyrrolic, c2-N doped pyrrolic). **(C)** Average potential of different numbers of Li intercalation in the graphitic, pyridinic, and pyrrolic defect structures of graphene. From Han et al. (2018) with permission from Royal Society Chemistry.

Lithium-ion batteries (LIBs) power many of today's electronics, and are widely used in electric vehicles and portable electronic devices (Armand and Tarascon, 2008; Magasinski et al., 2010; Zhong et al., 2015; Lu et al., 2018). It is urgent to develop high power density and low cost anode materials for lithium-ion batteries. Compared to the transition metal oxides that are easily swelled in electrolytes, carbon materials are preferred for current LIBs because of their low cost, high conductivity, environmental benign nature and inertness. NMCs are highly desirable since the rapid transport of solvated ions and charge can be guaranteed in the presence of mesoporous structure, and N-doping can improve the Li storage capability via the coordination interaction. The introduction of electronegative N (i.e., 3.5) affords a large number of active sites for Li-ion adsorption. In this regard, NMCs with high N content and optimized mesoporosity are expected to show high energy and power density in LIBs. Using N-rich MOF as the template, N-doped carbon honeycomb (NCH) structure assembled from 2-D mesoporous nanosheets (3.7 nm) was fabricated by a tandem thermal annealing and acid etching strategy (Han et al., 2018). These mesopores can facilitate the electrolyte transfer because the pores and interconnections provide favorable pathways for ion penetration and diffusion. The existence of N atoms produces a large number of defects in the carbon-based material, which may create more active sites for Li intercalation. Owing to the synergism between porous 2-D nanosheets and N dopants, the NCH exhibits a high reversible capacity of 609 mAh g^−1^ within 500 cycles (Figure 7A). The high structural integrity of the NCH after long-term cycling tests was confirmed by SEM observation and mapping. The structure has no obvious change after charging-discharging process. And it is found that the pyridinic N plays a more significant role than other N species in the increase of reversible capacity. To verify the role of pyridinic N during the charge/discharge process, first-principle calculations were performed to study the influence of N-doped graphene in LIBs. It is demonstrated that N doped in pyridinic graphene exhibits a stronger Li adsorption than others (Figures 7B,C), and the high specific capacity of NCH may be attributed to the high content of pyridinic N in sample.

**Figure 8:**
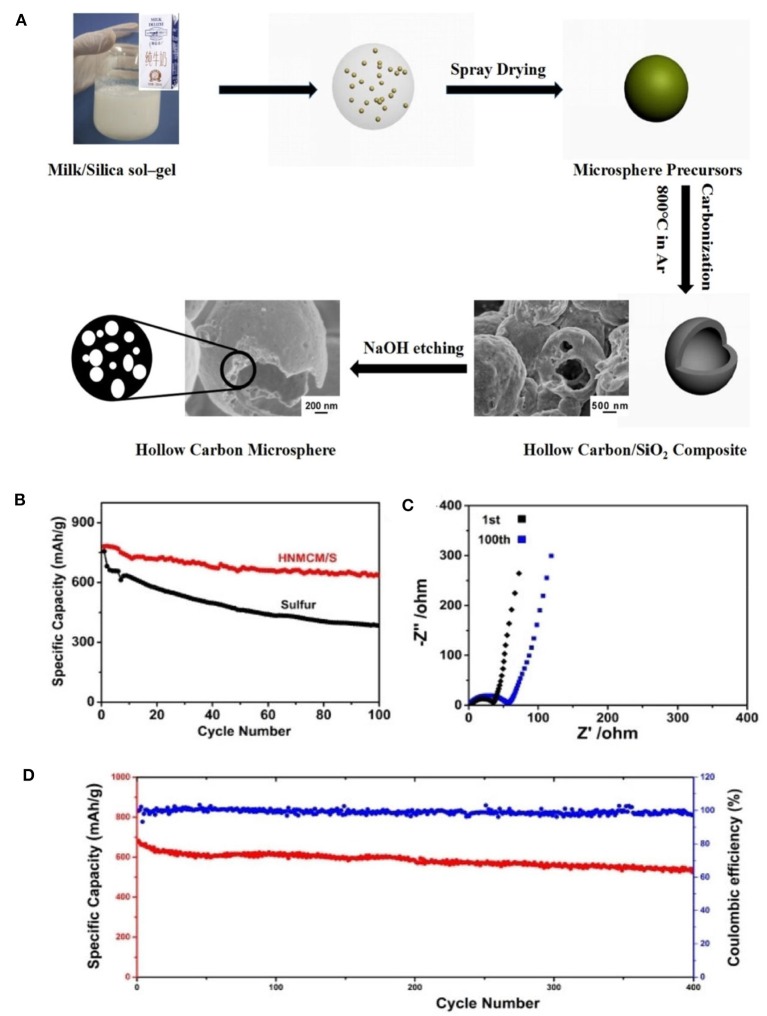
**(A)** Schematic illustration of fabrication of hollow carbon/SiO2 composites and hollow N-doped mesoporous carbon microspheres by spray drying method. **(B)** Comparison of cyclic performance of HNMCM/S composite and pristine sulfur electrodes at a rate of 0.5 C. **(C)** Electrochemical impedance spectra of HNMCM/S cathode before and after l00th cycles. **(D)** Long-term cyclic performance at a rate of 1 C. From Luo et al. (2018) with permission from Royal Society Chemistry.

**Figure 9:**
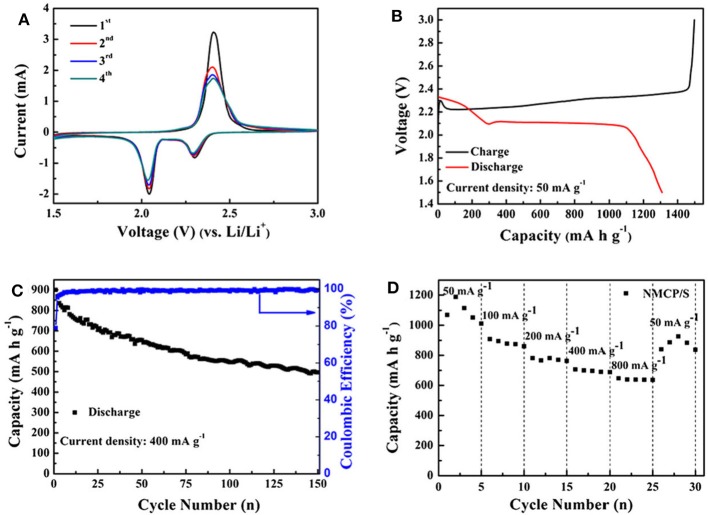
NMCP/S electrochemical performance. **(A)** CV curves, **(B)** charge/discharge profiles at 50 rnA g^−1^ **(C)** cycling performance at 400 rnA g^−1^, and **(D)** rate performance at 50 rnA g^−1^ From Zhang et al. (2019) with permission from SpringerLink.

As an ideal alternative to LIBs, lithium-sulfur batteries (LSBs) have received intensive attention recently due to the natural abundance of sulfur and high energy density (Manthiram et al., 2015; Peng et al., 2015; Wild et al., 2015). However, their commercialization was hindered because of poor conductivity and high solubility as sulfur was introduced. Such a problem can be overcome as N species are introduced, since the chemical bond between N-doped carbon host and polysulfide intermediate is formed, which could lead to a higher specific capacitance, rate capability and longer cycle life. And the mesopores facilitate the trap of soluble polysulfides in the sulfur electrode. Via a simple spray drying method (Figure 8A), a hollow N-doped mesoporous carbon microsphere (HNMCM) was prepared using outdated milk as the carbon and nitrogen source, showing a high specific surface area of 872 m^2^ g^−1^ (Luo et al., 2018). The resultant HNMCM delivers a higher cyclic stability than that of the pure sulfur cathode, showing an initial discharge capacity of 781 mAh g^−1^, which decreased to 634 mAh g^−1^ after 100 cycles at a rate of 0.5 C (Figure 8B). This can be ascribed to the excellent electronic conductivity, mesoporous structure of HNMCM, and the good dispersion of sulfur within the pores of HNMCM. Electrochemical impedance spectroscopy (EIS) measurement shows that the semicircle diameter of the HNMCM/S cathode moderately increased after 100 cycles (Figure 8C), indicating an increased charge transfer resistance of the cathode, due to the irreversible deposition of insoluble Li_2_S_2_ and Li_2_S after long cycles. More importantly, HNMCM/S cathode shows a capacity retention of 542 mAh g^−1^ after 400 cycles even at a high discharge rate as high as 1 C (Figure 8D). The N-doping could create the favorable chemisorption sites for lithium polysulfides to improve electrochemical performance of LSBs, and helps to increase the electrical conductivity of the material. The large void space in HNMCM is beneficial for the encapsulation of sulfur with a large loading. So the excellent cyclic stability and rate capability can be ascribed to high conductivity and unique hollow microsphere structure of HNMCM. Later, Zhang et al. (2019) developed ZIF-8 derived N-doped mesoporous carbon polyhedron (NMCP) as an efficient host. Upon sublimating sulfur into NMCP, the resultant NMCP/S delivers a high initial capacity of more than 1,300 mAh g^−1^ (Figures 9A,B). A long-term cyclic performance of the NMCP/S composite was observed at 400 mA g^−1^ (Figure 9C). And the rate capability of NMCP/S electrode at various current densities from 50 to 800 mA g^−1^ demonstrates an extraordinary rate performance (Figure 9D). The excellent properties of NMCP/S can be attributed to the mesoporous carbon polyhedral structure, which ensures the rapid diffusion of electrons and ions, and provides a large free space for the volume expansion of sulfur in the process of repeated charging and discharging. Meanwhile, the presence of N species can enhance the conductivity and interaction between the carbon host and the polysulfide guest via chemical binding and suppress the shuttle of polysulfides. As a result, the NMCP/S composite exhibits excellent stability and rate performance.

### Supercapacitors

**Figure 10:**
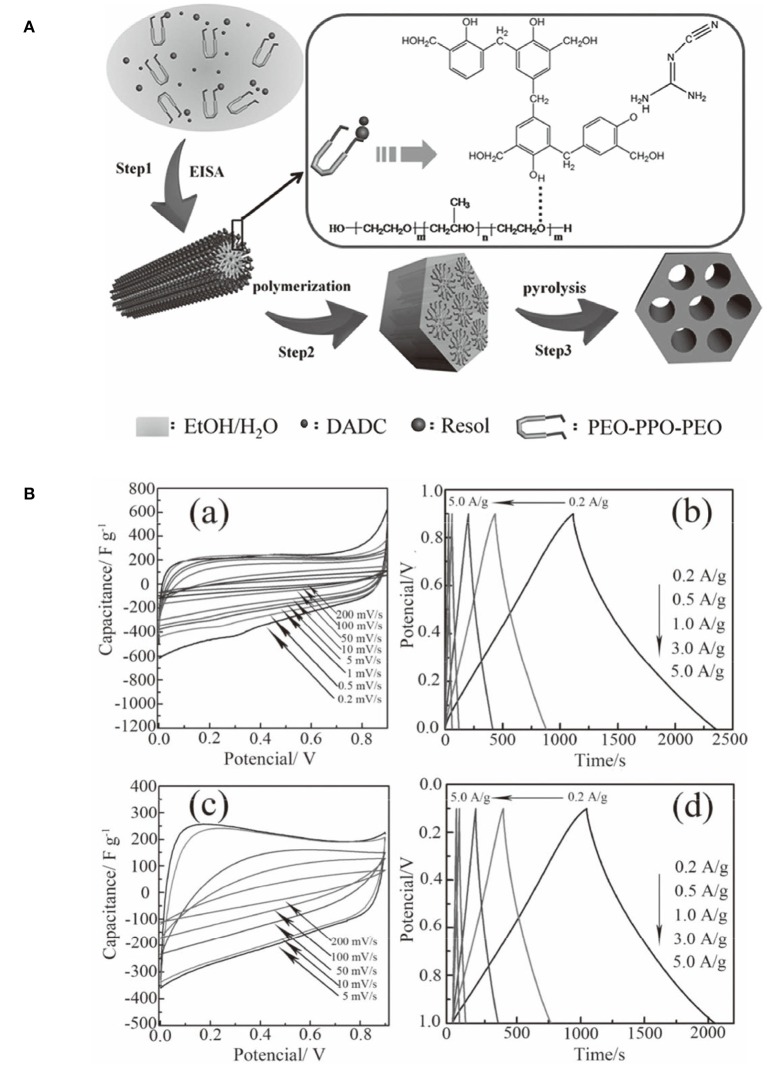
**(A)** The formation process of ordered N-doped mesoporous carbon from a one-pot assembly method using dicyandiamide (DCDA) as a nitrogen source. From Wei et al. (2014) with permission from Wiley. **(B)** Electrochemical performance of the sample H-NMC-2.5 using a three-electrode cell: cyclic voltammograms at different scan rates in (a) 1 M H2SO4 and (c) 6 M KOH; and galvanostatic charge/discharge curves at different current densities in 1 M H2SO4 (b) and (d) 6 M KOH. From Wei et al. (2014) with permission from American Chemical Society.

**Figure 11:**
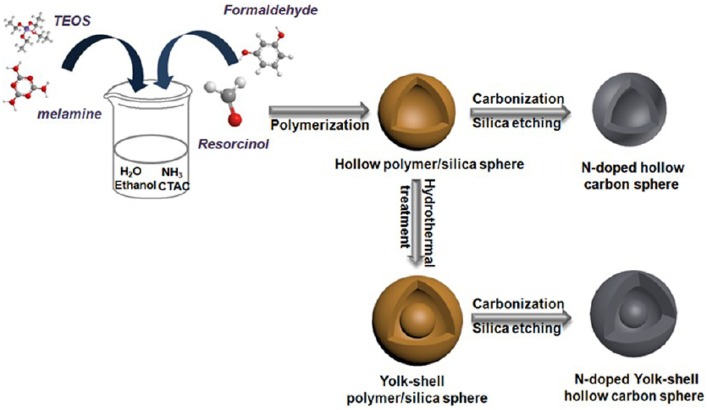
Synthesis of N-doped hollow-structured mesoporous carbon spheres. From Liu C. et al. (2016) with permission from ScienceDirect.

Mesoporous carbons have been widely used for electrode materials for electrochemical double-layer capacitors (EDLCs) based on the electrostatic interaction. The modification of carbon materials with N moieties, that can impart the acid/base properties to the carbon, typically contributes the extra pseudocapacitance from the striking redox process of the surface heteroatom functionalities. The enhanced capacity originates from the surface polarity, electrical conductivity, surface basic sites, and electron-donor tendency, while maintaining the superb cycle ability. However, it is still challenging but highly desirable to synthesize NMCs as high-performance electrodes for supercapacitors. An ordered mesoporous carbon was prepared using resol/dicyandiamide as the carbon/nitrogen source, F127 as the soft template via an evaporation induced self-assembly process (Figure 10A). The obtained N-doped ordered mesoporous carbons have tunable mesopore size ranged from 3.1 to 17.6 nm, high surface area (>450 m^2^ g^−1^) and N content (13.1%) (Wei et al., 2013). The resultant NMC shows high specific capacitances of 262 F g^−1^ (in H_2_SO_4_) and 227 F g^−1^ (in KOH) at a current density of 0.2 A g^−1^ (Figure 10B), which are much better than that for the mesoporous carbon FDU-15 without N modification (110–130 F g^−1^) (Wei et al., 2013). The excellent performance of NMC is as a result of high surface area and high N content, as well as highly accessible mesopores. Larger surface area, N-doped hollow mesoporous carbon nanospheres are fabricated by a “silica-assisted” route (Figure 11). The morphological and pore structures of these carbon nanospheres can be tailored by varying the ammonium concentration. The large surface area (2,464 m^2^ g^−1^) and mesoporous structure allows a large amount of electrical charge to accumulate on the electrode/electrolyte interface. The small particle size provides a large additional pseudo-capacitance because it shortens the ion transport length and makes ion diffusion in the carbon nanospheres easier and thereby enhanced the capacitance. On the other hand, the enhanced capacitance also can be contributed to the N functionalization, that could enhance the electrical conductivity and electrolyte solution wettability of the carbon materials. Owing to the hollow mesoporous structure and N functionality, these NMC nanospheres manifest excellent supercapacitor performance as the electrodes with high capacitance up to 240 F g^−1^, favorable capacitance retention (97.0% after 5,000 cycles) and high energy density of 11.1 Wh kg^−1^ (Liu J. et al., 2016).

**Figure 12:**
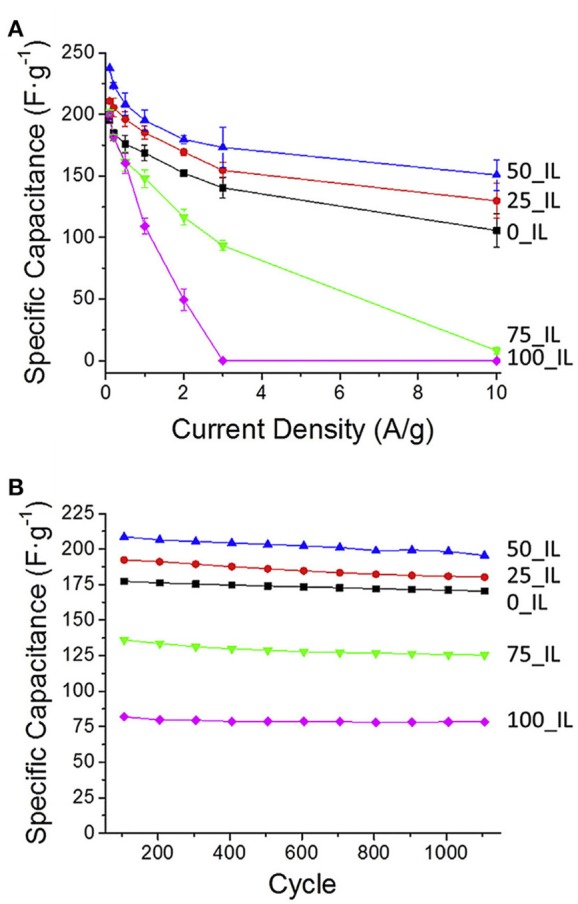
**(A)** Average specific capacitance of the carbon materials over a range of rates. **(B)** The specific capacitance of the carbon materials over ca. 1,100 cycles at 1 A g^−1^. From Wilson et al. (2015) with permission from ScienceDirect.

**Figure 13:**
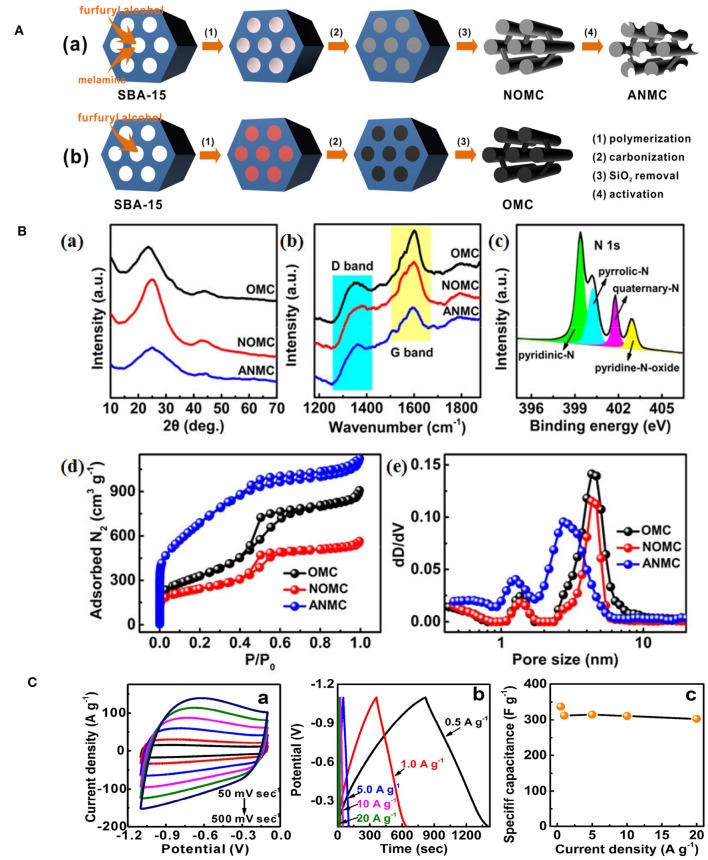
**(A)** Schematic illustration for the synthetic procedures of (a) ANMC and (b) OMC. From Chen et al. (2017) with permission from ScienceDirect. (**B**,a) wide-angle XRD patterns and (b) Raman spectra of OMC, NOMC, and ANMC samples; (c) XPS N1 s spectrum of ANMC sample. (d) Nitrogen adsorption-desorption isotherms and (e) the corresponding pore size distributions of OMC, NOMC, and ANMC by DFT method. From Chen et al. (2017) with permission from ScienceDirect. **(C)** Electrochemical capacitive behaviors of ANMC: (a) CV curves at scan rates of 50–500 mV sec^−1^, (b) GCD curves at current densities of 0.5–20 Ag^−1^, and (c) The relationship of specific capacitance vs. current density. From Chen et al. (2017) with permission from ScienceDirect.

Hard-templating approach was also used to prepare NMCs for supercapacitors. Using silica colloidal hard-template, phenol-formaldehyde and 1-ethyl-3-methylimidazolium dicyanoamide as carbon and nitrogen precursors, well-controlled NMC was constructed, bearing a high specific surface area of 1,070 m^2^ g^−1^ (Wilson et al., 2015). Compared with N-free mesoporous carbons, the specific capacitance increases over 40% as 50% of IL was utilized, and the maximum capacitance is 237 F g^−1^, an 18% improvement over the undoped sample (Figure 12A). This NMC can be cycled 1,000 times in the ionic liquid electrolyte, having an average specific capacitance of 202 F g^−1^, that presents a 16.5% increase over the average specific capacitance of undoped sample (Figure 12B). It is found that the interconnected mesoporous structure and N-doping are responsible for the enhanced capacitance and stability. Xi's group fabricated an activated N-doped mesoporous carbon, ANMC, by a hard-templating method using SBA-15 as the template followed by KOH activation (Figure 13Aa). Ordered mesoporous carbon (OMC) was also prepared by the similar procedure without melamine added and KOH activation (Figure 13Ab). XRD investigations reveal that the (002) peak of N-doped samples shifted to higher 2θ value. And the decreased interplanar spacing in NOMC and ANMC corresponds to the change of electron localization and the promotion of interlayer action in the N-doped structure (Figure 13Ba). The graphitization level, estimated by the I_D_/I_G_, was 0.519, 0.595, and 0.816 for OMC, NOMC and ANMC, respectively (Figure 13Bb), which suggests that the ordered structure was destroyed by the chemical activation. As for the N dopants, pyridinic N and pyrrolic N contribute the pseudocapacitive interactions, while the quaternary N and pyridine N oxide may facilitate the electron transfer in the carbonaceous skeleton, therefore enhancing the conductivity (Figure 13Bc). What is more, the N doping favored the wettability between electrolyte and electrode materials, consequently promoting the formation of electrical double-layer at the interface. As for the pore structure, ANMC shows an increased N_2_ adsorption as compared to OMC and NOMC (Figure 13Bd), corresponding to a higher surface area (2506 m^2^ g^−1^) and pore volume (1.74 cm^3^ g^−1^). The pore size distributions in Figure 13Be show much more pores in the range of 2–4 nm for ANMC. The mesopores centered at 2–4 nm proves to be suitable for the rapid transfer of electrolyte ions. The enhanced charge and electron transfer rates could increase the number of usable charge storage sites (Chen et al., 2017). The unique pore structure of ANMC is favorable for achieving superior electrochemical performance. As expected, this ANMC exhibits efficient electron transfer with the scan rate from 50 to 500 mV s^−1^ (Figure 13Ca), and a super charge/discharge ability with the current density range of 0.5–20 A g^−1^ (Figure 13Cb). A superb specific capacitance of 336.9 F g^−1^ at 0.5 A g^−1^, that was still kept at 302.9 F g^−1^ at the current density of 20 A g^−1^, 89.9% of the value at 0.5 A g^−1^ (Figure 13Cc). The high rate capability of ANMC can be ascribed to the rapid transport of electrons on carbon networks and the fast diffusion of electrolyte ions in the porosity. Moreover, it is found the capacitance of NMCs is N content dependant. For example, as the D-glucosamine was infiltrated into the pores of SBA-15 and was pyrolyzed from 900 to 700°C, the N content was declined as the pyrolysis temperature was elevated. The sample pyrolyzed at 700°C shows a higher specific capacitance, as well as better cycle ability (95% of the initial capacitance after 5,000 cycles) in 6 M KOH, mainly owing to the incorporation of more active N species at a lower temperature (Fang et al., 2018).

Direct pyrolysis of a *pre*-formed N-containing carbon source was also employed to prepare NMC electrodes, which can minimize the high cost with templates used. In the primitive study, polypyrrole, polyaniline, and polyacrylonitrile were widely used as the precursors to prepare NMCs, however, poor solubility and metal contaminant from the catalyst made such a synthesis less feasible. A NMC was recently prepared by one-pot conversion of biopolymeric milk powder and KOH mixture without templates. The resultant NMC has a high surface area of 2145.5 m^2^ g^−1^ and a pore volume of 1.25 cm^3^ g^−1^, and the N content is 2.5%. Due to the high porosity and active N functional groups, the NMC exhibits a high specific capacitance of 396.5 F g^−1^ at the current density of 0.2 A g^−1^ and excellent stability of 2,000 cycles at 50 mV s^−1^ (Jia et al., 2016).

**Figure 14:**
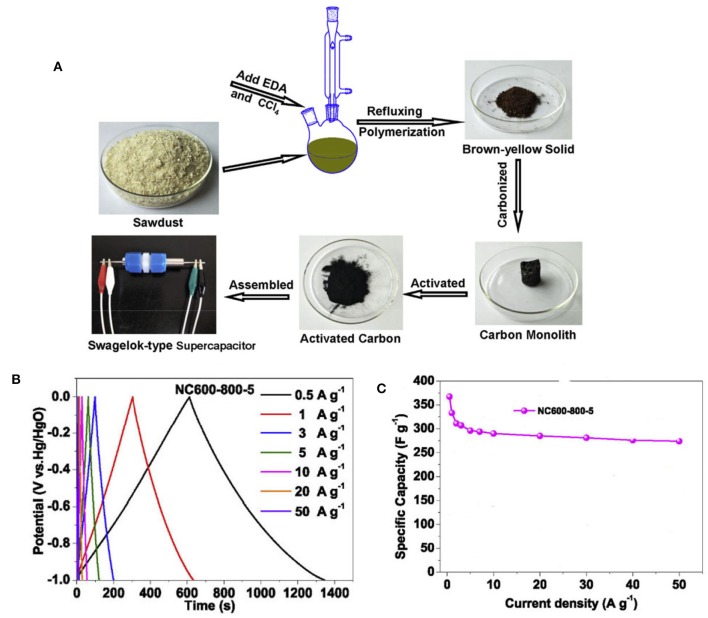
**(A)** Schematic preparation of the nitrogen-doped activated carbons, **(B)** CV curves at different scanning rates for NC600-800-5, and **(C)** the specific capacitances at different current densities for NC600-800-5. From Guo et al. (2019) with permission from ScienceDirect.

Recently, inedible biomass of waste sawdust has been used as the carbon source to prepare NMC supercapacitor electrode, with ethylenediamine and carbon tetrachloride as the nitrogen source (Figure 14A). The NMC prepared by further activation with potassium hydroxide can improve its surface area and increase the electric double-layer capacitance, while the pseudocapacitance can be improved by heteroatoms. Correspondingly, a high specific capacitance of 367 F g^−1^ is reached at 0.5 A g^−1^, and 96.7% of the initial specific capacitance is kept after 10,000 cycles (Figures 14B,C). It is found that the introduction of N (graphitic-N) could not only enhance the electrical conductivity, but also improve the specific capacitance of carbons by increasing the wettability of carbon surface. This is because increase of wettability can improve the utilization efficiency of surface area and further improve the electric double layer capacitance. Thus, it is demonstrated that the introduction of N element into carbons is particularly important to improve the electrochemical performance of NMC materials (Guo et al., 2019).

## Conclusion and Outlook

Much effort has been made to develop NMCs within high surface area and superior N doping content as reduction catalysts and energy storage materials, however, the present NMCs are still far from satisfactory for applications. These NMCs are mainly synthesized through hard-/soft-templating, or template-free approaches. And the resultant NMCs prove to be superior catalysts for oxygen reduction reactions, electrochemical synthesis of hydrogen peroxide, nitro compound reductions, and electrodes for Li-ion/sulfur batteries and supercapacitors. Although various studies demonstrate that superior mesoporous structure are beneficial to mass transfer and active site dispersion, and the content of graphitic/pyridinc N affects the performances, it is considered that the synergism between mesoporous structure and N-doping plays a significant role. Much research is further needed to produce other efficient NMCs via an inexpensive, facile method for wider applications. To achieve this, several points should be addressed as follows.

1. The synthesis of cheap NMCs via facile and effective methods is of great importance. Most of the present synthesis is not very efficient or has a tedious procedure, therefore, development of simple, effective and scaled-up synthetic strategy for new NMCs will be viable to achieve practical applications. The direct conversion of crystalline N-containing MOFs proves to be highly efficient for the synthesis of NMCs, that will be applicable in practice if cheap N-containing struts can be found.
2. Recent reports have focused on NMCs with enhanced performances toward catalytic reduction reactions and energy storage. It is noteworthy that the present NMCs are mainly used as ORR catalysts and supercapacitor electrodes, and their utilization in electrochemical production of hydrogen peroxide and nitro compound reduction, as well as used as electrode materials for Li-ion/sulfur batteries should be strengthened. Furthermore, some investigations about N-doped carbons show the improved catalytic properties in the electrochemical oxygen evolution reactions (OER) and C-H bond activation reactions, like oxidation of ethylbenzene and cyclohexane (Yu et al., 2011; Gao et al., 2013). It is anticipated to develop NMCs for OER and C-H bond activation reaction catalysts, but not limited, for wider applications.
3. For NMC catalysts, in order to elucidate the relationship between nitrogen bond structure and ORR performance, some studies have been carried out, but the precise relationship between catalytic activity and N species is still not clear. Total or active N content affects the catalytic performance of NMCs in ORR, or the species of graphitic or pyridinic N play a significant role? So deeper investigation is thus required, besides N elemental analysis and X-ray photoelectron spectroscopy (XPS), advanced techniques, such as X-ray absorption near-edge structure (XANES) and electron energy loss spectroscopy (EELS), should be employed to explore the N dopants, to develop highly efficient NMCs with well-defined N species finally. On the other hand, the mechanism of activity and capacitance enhancement for most NMCs is absent. It is considered that the positively charged sites could facilitate the side-on O_2_ surface adsorption, that were created by breaking of the electroneutrality of graphitic materials (Yang et al., 2011). Density functional theory calculations demonstrate that the spin density governs the catalytic activity (Zhang and Xia, 2011). Currently it is found that as the N dopants were introduced, higher strains were generated at the edges, thus facilitating charge localization and related oxygen chemisorption, leading to an enhanced ORR activity (Jin et al., 2012). The above inconsistent conclusions prove the complications of mechanism for enhanced activity, and it may be varied for diverse N dopants and reactions. In this case, deep investigations about the N dopants and the performance, as well the reaction mechanism can be referred in the future.

## Author Contributions

YY and SH wrote and modified this paper. Other authors searched literature and wrote the references.

### Conflict of Interest

The authors declare that the research was conducted in the absence of any commercial or financial relationships that could be construed as a potential conflict of interest.
